# A Letter to Sir Benjamin Brodie, F.R.S., &c., Containing a Critical Enquiry into His 'Lectures Illustrative of Certain Local Nervous Affections.'

**Published:** 1841-07

**Authors:** 


### ART. VII.

*A Letter to Sir Benjamin Brodie, F.R.S., &c., containing a critical enquiry into his 'Lectures illustrative of certain Local Nervous Affections.'* By WILLIAM GOODLAD, M.R.C.S. &c.—*London*, 1840. 8vo, pp. 154.

IT must be confessed that, notwithstanding the advance which has been made on the subject, we are still very much in the dark respecting the pathology of what are, unsatisfactorily enough, termed nervous affections. The brain, the spine, the nervous system generally, the uterus, the stomach, and chylopoietic system, are by different individuals regarded as the centre of the symptoms termed nervous, whilst others imagine them to reside in a certain hereditary or acquired constitutional disposition, &c. And the treatment is vague and various in correspondence with these varieties of opinion. Sir Benjamin Brodie performed a valuable service in insisting on the importance of studying "each individual case pathologically, endeavouring to trace the symptoms to their true origin." Those who have endeavoured to systematize these diseases in accordance with any recognized nosological arrangement must have felt the propriety of this direction, however laborious and, in many instances, unsatisfactory its application. For it is a truth of which any reflecting person must be readily convinced, that a careless and immature classification, requiring as knowledge advances to be new modified and changed, is productive of far more trouble than the consideration of individual cases, either quite independently one of the other, or only in such connexion as, without any forced relationship, they will bear. The lectures of Sir Benjamin Brodie, which are reviewed in the volume before us, were reviewed by ourselves in our Seventh Number, and are doubtless well known to the profession generally. The lectures contain a theory of local nervous affections, of what its author terms local hysterical affections, an attempt to explain these pathologically, and directions for general management and surgical or medical treatment, such as have been derived from the author's very extensive experience.

Before proceeding to a consideration of the matter of Mr. Goodlad's volume, we are compelled to state that, although he expresses regret if he should "even in the least degree have deviated from that course and courtesy which a love of truth and his profession dictates," the volume before us is sadly characterized by his misrepresentations of the observations of Sir Benjamin Brodie. We shall not hesitate hereafter to speak in terms of commendation of anything which appears to us to merit praise, or to condemn views against the correctness of which Mr. Goodlad has adduced sufficient evidence; but the same sense of justice compels us, in the most unqualified manner, to condemn the garbled representations which are made of the views of Sir B. Brodie. We are far from wishing to accuse Mr. Goodlad of any intention to make the lectures of Sir B. Brodie appear absolutely absurd; but a severe censure is merited by any one whose incompetence to weigh the value of words, or whose bias against the opinion of another leads him to commit the errors which especially belong to the first few pages of Mr. Goodlad's volume. In illustration of the above remarks we refer, among many others, to p. 6; and we hesitate not to say that we know no page in any other volume so completely full of truth in the letter and of untruth in the sense as that referred to.

There is too great a disposition on the part probably of everybody to escape from a difficulty in pathology under the cover of a name. Such may be said of the general use of the term hysteria. But in every attempt to classify with greater correctness the diseases commonly termed hysterical, there is great danger that little more may be effected than to change one name which is inappropriate for another almost if not quite as objectionable. Speaking of Sir B. Brodie's employment of the term hysterical, Mr. Goodlad says, "If, as I hope to show, in hands like yours this doctrine leads to false conclusions and dangerous because inert practice, if under this name disorganization creeps on whilst the practitioner is waiting, and if your lectures establish this fact, it requires little more to point out the necessity of confining this term to its legitimate meaning," &c. It is needless for us to make any detailed reference to the lecture referred to. Mr. Goodlad thinks that there is great objection to regarding many of the cases therein mentioned as hysterical, and we in some degree coincide with him in the opinion. A case related by Sir B. Brodie (p. 67 of the Lectures,) as hysterical, in which there had been retention of urine, and a diseased condition of the bladder was found after death, is thus commented on by Mr. Goodlad:

"If this case had been set up as a beacon to warn your pupils against the danger of the doctrine of hysteria, it could scarcely have been more instructive; and it is precisely because it furnishes an alarming proof how, even when united with the greatest intelligence, this theory leads to fatal consequences, that it becomes an imperative duty to examine it. And hence it is that when you say 'there was no sign of mortification but the black colour,' and infer that the colour was not dependent on that cause because there was 'no fætor,' it seems right to remind you that in strangulated hernia there is often 'no fætor,' though the intestine is found 'almost black;' and that where it is so, and especially where 'the coats are easily separated from each other,' no surgeon has ever questioned either that mortification already existed, or that the part was on the verge of mortification." (p. 17.)

But whilst we think with Mr. Goodlad that this case exhibits something more than hysteria, we cannot draw from it all the inferences which he would desire to deduce. We should consider it as either an exception to a general rule, or as a case about which a wrong opinion had been expressed. But as regards the general doctrine and practice recommended in these cases, we must remember that Sir B. Brodie says, "Females who labour under hysterical retention of urine, if left to themselves, *usually recover in a short space of time, sometimes almost suddenly; but if the catheter be employed their recovery may be protracted for an indefinite period.* We may lay it down as a general rule, that in these cases the catheter should not be had recourse to; and the only exceptions to it are in those extreme cases in which actual paralysis has taken place, and the bladder is likely to become diseased, if not artificially relieved." We have marked in italics that part of the above quotation which especially applies to our present subject. Sir B. Brodie observes, when speaking (p. 36, Lectures,) of local pains apart from local disease, "that where there is that state of the general system, whatever it may be, which produces the phenomena of hysteria, it is not uncommon for a particular joint to be affected with pain and morbid sensibility, such as may lead a superficial observer to believe that it is the seat of some serious local disease, although no such disease in reality exists." Our readers are doubtless acquainted with Sir B. Brodie's endeavour to explain the phenomena of hysteria. Mr. Goodlad observes on the above quotation that "it may be assumed as an axiom that will bear universal application, that wherever there is continued pain, there is increased vascular action somewhere, the discovery of the seat of which becomes the duty of the practitioner." (p. 29.) And he further remarks that in none of the cases mentioned by Sir B. Brodie, was any enquiry made with a view to ascertain whether there existed any cause of irritation at the *origin of the nerves* which were the seat of suffering, expressing at the same time his confident belief that "if the enquiry had been made, an adequate cause would have been discovered." It will thus be evident that the author's object is to explain the phenomena of these cases which are attributed to hysteria on the theory of some morbid condition of the circulation in the spinal cord. And although there may be no great novelty in this view of the subject, we do not think that time will be lost in referring to it for a short time.

Our readers will excuse our passing over much of the volume before us which is merely devoted to an examination of Sir B. Brodie's views, especially as much which is merely theoretical is the subject of criticism. Indeed, our own opinion respecting what are termed nervous diseases is one which is rather eclectic than attached to any particular system; and we find in the endeavours of one pathologist after another to localize these diseases in particular organs or systems sufficient ground to believe that they are all more or less wrong, although each of them may possess a portion of the truth. We have no doubt that Sir B. Brodie's volume contains not a little of this. We know likewise that there is some of it in the volume before us; and if we can find in it anything which is new to many of our readers, or which may refresh the memories of others with views which they have long in various degrees entertained, our purpose will be sufficiently answered.

Mr. Goodlad considers that the sympathies manifested in some of Sir B. Brodie's cases (such, for instance, as the production of pain in the ankle by acidity in the stomach,) are best explained by supposing the cause to exist at the origin of the nerves of the affected parts in the spinal canal. He believes that the ganglionic system is of little use in regulating sympathetic disease. "I shall proceed therefore," he says, "to prove that the different portions of the spinal cord have the power of *creating* disordered action like the brain, and that this may arise in any part of it independent of any other; the effects of which are observed only at the extremities of those nerves connected with it." The first case, which is one of great interest, we must notice at some length. It is that of a female, aged fifty-two, who was violently convulsed in the lower limbs, February 1819. Every treatment which was thought likely to be useful was employed without effect, until, whilst examining the region of the kidneys, "on pressing upon the neighbouring spinous process of the vertebræ, the patient cried out 'there,' and immediately a strong convulsive action commenced, and pursued its usual course." The pressure was repeated, and each time with a repetition of the paroxysm. Leeches were applied, and the convulsive action ceased. In the following October the convulsions returned, but with much more violence, and were again in the same way relieved. In June 1823, the same happened, the convulsions, however, being much more general and violent, and the spinal affection in a higher part of the column. "The slightest pressure brought on a fresh attack; twenty leeches were again applied, each leech producing a similar effect as it pierced the skin, but effectually relieving her by the time they were filled, after which she obtained several hours' uninterrupted sleep, and had no return. No medicine of any kind was administered." The following year the attacks returned, and were relieved in the same manner. She continued well until 1839, when she suffered a similar attack. In this she died. In making an examination after death—

"The processes of the vertebræ of the back, and the whole of the spinous processes of the vertebræ of the neck were divided.......The cord exhibited in detached portions of its surface very considerable vascularity.........But although some portions of this, the posterior aspect of the dura mater, were very vascular, this was by no means universal, some portions of that membrane presenting a natural appearance. The medulla was then cut across, about the eighth dorsal vertebra, and was raised very carefully from its situation.. ... The anterior surface of the dura mater, covering the spinal column, presented one entire vascular mass, and the connexion between it and the membrane lining the canal was more intimate than natural. This great vascularity was most striking and most extensive on the external surface of that membrane, where flocculent masses of a bright scarlet were seen floating from it. In some parts, this outer surface being removed, the dura mater, properly so called, was observed of a paler colour beneath it, whilst at those portions of the surface where the nerves are detached, or at the point of union between the nerve and the spinal cord, to speak more correctly, the vascularity was greatest, and congeries of vessels were seen extending from nerve to nerve on each side....... The dura mater covering the medulla oblongata was ecchymosed, like the conjunctiva after a blow, and was scarcely less vascular.........Returning to that portion of the canal from which the spinal cord had been detached, there were observed distinct patches as if of extravasated blood..........This vascularity was between the theca and bodies of the vertebræ or of the intervertebral substance." (p. 63, et seq.)

Mr. Goodlad remarks,

"It is only when the disease of the spinal column extends by continuous sympathy to the ligaments that pain on motion is experienced, and only when it extends to the origin of the sentient nerves that there is an increase in perception amounting to pain in other parts. Whether the disordered action is confined to the muscles of one part or extends to many depends on the extent of the original cause; but innumerable cases may be related where distant and distinct parts of the column labour under complaint, the intermediate portions being healthy, and where impressions made at the extremity of the nerves connected with one of these portions will be transmitted to and produce disordered feeling at the extremities of the others." (p. 81.)

Different states of disordered feeling are explained by supposing disordered vascular states at the orifice of the nerves. After referring to the opinion of Sir H. Halford, "that tic douloureux of the face arises from irritation of the nerves, occasioned by a portion of dead or carious bone," Mr. Goodlad continues:

"It requires only the admission of this fact and an acknowledgment of what no one will deny, that a similar cause operating near the source of the spinal nerves will produce the same or a like effect, and all the phenomena of what Sir

B. Brodie calls hysteria are accounted for; and we are justified in believing that the disorders in the cases quoted by Sir B. Brodie have this origin, until it is proved by investigation that such a cause does not exist, and unless it can be shown also that there are some symptoms which such a condition will not explain." (p. 83.)

We give the above as Mr. Goodlad's views, but not as reasoning which is to us quite satisfactory. In deciding any such question it is not necessary merely to say that the supposed cause would be sufficient to produce and to afford an explanation of all the symptoms. We must have the evidence of the actual existence of such a state of the spinal cord, and the proof of the connexion between this and the symptoms characterized as hysterical, before we can absolutely determine the question. In the meanwhile, however, our own opinion and that of very many others tend to the belief that such is actually the case. But whatever objections may be made to the views of Sir B. Brodie on the theoretical question, we feel compelled to defer to his judgment when decidedly offering an opinion on a point of practice. And on this point we should refer again and again to Sir B. Brodie's observations on certain heroic modes of treatment which are too often adopted in accordance with the views of spinal irritation or inflammation. Blisters, leeches, various forms of counter-irritation, and remedies calculated to exhaust power are often employed in cases of this character where the proper mode of remedying the morbid state of the spine, even supposing it to have been one of increased vascularity, would have been such general means as are best calculated by means of diet, exercise, &c. to benefit the general health. The following case, which occurred in our own practice, is one out of many which we, in common with every experienced practitioner, could adduce in favour of Sir Benjamin Brodie's practical views.

A lady, very excitable both morally and physically, had been for some time under our care, affected with what is usually termed *spinal irritation.* She benefited under the usual means, and was in very good health, when, in consequence of long-continued fatigue and great anxiety, she was attacked with faintness. To this there succeeded some reaction, great pains in the head and limbs, and some spasmodic motions in them; and on examining the dorsal vertebræ three of them were found acutely painful on the slightest pressure. Here we thought was a case for leeching the spine, which we accordingly proceeded immediately to do. No local benefit was derived from this treatment, but in about two hours afterwards the spasms became of a tetanic character, accompanied with symptoms of exhaustion which we were disposed to attribute to the loss of blood in a state of debility. The progress of this case was marked by the frequent recurrence of tetanic symptoms, which nothing but large opiates would allay; but the amendment which took place in it commenced with the time that medicines and applications to the spine were disregarded, and attention was given, by diet, frictions, gentle exercise, &c., to the state of the general health.

We do not mean to infer from this that Mr. Goodlad's theory is necessarily wrong, but that Sir B. Brodie's remarks on treatment are worthy of much attention when he discourages the free use of means which in many hands might prove very injurious. It is quite consistent with analogy to suppose that a morbid vascular condition of the spine may exist in many hysteric cases, but which would require any treatment rather than local depletory means for its relief. It appears to us that we may not unfairly infer much respecting the vascular state of the spine, and the most appropriate means of relieving it, from that which we frequently witness in the various tunics composing the eye. No one in these days would look upon leeches, blisters, and remedies of this class, as the best means, under many circumstances, of restoring the distended vessels of these tunics to a healthy state of contractibility. And we must for our parts agree with Sir B. Brodie in the great advantage derived in many cases of the kind alluded to from the use of palliatives, e. g., belladonna plasters, &c. That they should be useful consists with the fairest reasoning and the best experience. It is quite legitimate to suppose that the existence of pain impairs the proper exercise of a function. Take away this pain and aid the system as much as possible in the exercise of its self-restoring power, and the utility of palliatives becomes sufficiently manifest. There is much ingenuity in Mr. Goodlad's reasoning, and we derive from it additional reasons for paying great attention to the condition of the spinal column in all cases of hysteria. But this is now a part of practice which nobody thinks of neglecting. Mr. Goodlad has some good observations on the reasons why appearances after death are not more frequently found in the spinal column, and on the peculiarities of its circulation which favour congestion of its vessels. We quote a part of these:

"A great portion of the medulla spinalis receives its blood from more numerous sources than the brain, each portion or bundle of nervous matter having its distinct nutrient artery sent off from the aorta, and each portion having also its returning veins. Disorders within the spinal canal are not only limited, like those of the brain, by this mode of circulation, but are still further confined, for the ligamentum denticulatum acts like a tentorium, and forms a line of separation by which each portion remains distinct from contiguous ones, either above or below it; and pressure, whether it arises from effusion or congestion, influences one part of the medulla and the nerves arising from it, although not extensive enough to influence other portions of that substance, or the nerves arising from other parts. In like manner one fasciculus of nervous matter suffers from inflammatory action without the others or any of them participating. In other cases inflammatory action, in which one nerve or one set of nerves participates, shall have gone through its stages, producing effusion and consequent pressure and paralysis of those nerves, whilst the circulation in another contiguous circle shall possess greater activity than is natural; and we have paralysis in one part and pain in another; loss of sensation and increased action of muscle in one individual, or loss of muscular power and increased sensibility in another; asthma and epilepsy in one case, asthma and hemiplegia in others." (p. 102.)

The great influence of certain conditions of the spine upon the heart is very ingeniously argued by Mr. Goodlad. Those who have paid any attention to the symptoms commonly ascribed to what is termed spinal irritation, know well how constantly the heart is affected. Mr. Goodlad says that he is now satisfied "that in passive dilatation of the cavity, the heart is to all intents a palsied muscle, and paralysis there, as in all other cases, takes place as a consequence of effusion into the spinal canal." We do not consider that this connexion is at all proved, though at the same time it does not appear unreasonable to attribute some cases of passive dilatation to some cause diminishing the nervous influence which is transmitted from the spine to the heart. The author's reasonings on this subject lead him to remark, on the importance of paying great attention to the state of the spine in apparent diseases of the heart, and not in these only, but in those of all other viscera ; and as our views entirely coincide with his on this subject, we shall be excused in making another extract :

"The knowledge of the existence and the progress of disease in different portions of the spinal medulla, and in which the nerves connected with those parts participate, simplifies the symptoms observed in a great variety of disorders, and gives a precision to practice which can only be obtained, I believe, by this knowledge. Thus, disorders of the uterine organs, and the chain of circumstances, numerous and extensive as they are, which connect these organs with other parts, can only be understood by the admission of corresponding local causes at the origin of nerves connected with them only through the spinal cord. . . The nerves of function in one organ, as the stomach, will labour under the same disordered action as those of the heart, and symptoms of indigestion will exist in such cases long before disorders of the heart are manifested, the latter being called into activity by the extension of the complaint in the spine from a severe cold ; by inflammation in the lining membrane of the air-tubes in the lungs, or by cold applied to the surface of the body, and vice versâ. Where such disorder does exist in different portions of the cord and in the nerves arising from it, very slight causes applied to the extremities of one set of nerves, will immediately operate on the organs connected with the other; and hence, on the approach of menstruation, we have not only pain experienced in those organs connected immediately with that function, but inordinate vomiting in one case, violent action of the heart, and violent action of the muscles connected with respiration in another. . . . In most cases of puerperal convulsions, so far as I have witnessed them, previous disorder may be traced, and its existence denoted, by tenderness at the origin of some of those nerves connected with the womb. The act of parturition, and the increased vascular action which is connected with it, are well calculated to give activity to a state of disorder already existing there, although not sufficient to overpower the ordinary functions of any part, without the additional assistance of such a cause." (p. 122.)

The above contains much matter for profitable reflection, and we have found that a mode of reasoning like this has generally appeared to afford us explanations of symptoms which are otherwise not easy to explain. Agreeing with Mr. Goodlad in much which he has advanced, and being ourselves disposed to think that the spinal cord has a most important share in the production of various symptoms termed hysterical, we do not however think that his views of treatment are quite correct. To subdue the inflammatory action which is supposed to exist in the spine, he thinks that caustic issues and rest will often be required, as well as other depletory local treatment; and cases are subjoined illustrative of the propriety of the practice. On this point we shall make a few observations, and with these conclude our remarks on the volume before us, which we do not hesitate to recommend to our readers as well worthy of their attentive perusal.

In a practical point of view, it is very necessary to employ the term *inflammation* in a precise sense. If we observe that which is commonly called inflammation, as it exists in the eye or skin in various states, and in both of which structures it falls more immediately under our observation, we learn that for this condition the most various and opposite treatment is required. At one extreme and in one constitution, local and general depletory measures are called for, and at the other a treatment of directly opposite character ; and between these extremes are other conditions requiring special management. The state of simple hyperæmia of a part is sometimes spoken of as inflammatory, although quite different from it, and having no tendency to the production of effusions such as characterize inflammation. Now, with regard to the cases which have been treated of by Sir B. Brodie and Mr. Goodlad, there is no evidence that inflammation, taken in the strict sense above referred to, is their uniform or common accompaniment. It is true that a case or two may be quoted in which the effects of inflammation have been witnessed after death, and apparently affording a rational explanation of the symptoms which existed during life; and it is further true that occasionally the gradual progress of these cases is such as to allow us to suppose that some disorganization may have taken place at the origin of nerves, or that their functions may be interfered with by deposits resulting from inflammation. But this is not the ordinary course of such cases; the history of them is such as does not justify the supposition of an actual inflammation existing in the spine. The symptoms are frequently intermittent, exist a long while, and are suddenly recovered from, and the completeness of recovery is not such as could be supposed to follow a long inflammatory action going on in the spine. And the treatment which has been found efficacious in the hands of many also militates against the idea of inflammation being a cause. We should be much more inclined to believe that a congested state of the spine existed than an inflammatory state, and such an opinion affords a more credible explanation of symptoms than the supposition of inflammation; and it likewise points out the mode of treatment which we should recommend, and explains its utility. We think that the phenomena of blushing may be usefully considered in relation to the present matter. This congestion is a result of moral and physical weakness, and it is a tolerably constant characteristic of the hysteric class of females. To cure it, we should not leech and blister or enjoin rest, but we should expose abundantly to fresh air, insist on regular and plentiful exercise, with sufficient intervals of perfect rest, feed with a most generous and nutritious diet, and, with but little apprehension in the majority of cases, of failing in effecting a cure. We think that if there be anything wrong in the circulation of the spine in the cases alluded to, it is generally a congested state arising from debility or exhaustion, to be explained by some hereditary peculiarity, some bad habit or mode of education, some series of practices inimical to health, some moral or physical cause which, in by far the majority of cases, produces a condition, the proper remedy for which is the system which a boxer would adopt were he training for the exercise of his calling, modified according to the peculiar circumstances of the case. Daily experience is teaching us the omnipotence of such means in the treatment of various forms of disease; and without meaning to assert that the cases do not occur which require the treatment recommended by Mr. Goodlad, we are well assured that the class of complaints to which Sir B. Brodie has applied the term hysterical are not to be so dealt with.

